# Breast cancer in elderly patients: a clinicopathological review of a Nigerian database

**DOI:** 10.3332/ecancer.2022.1484

**Published:** 2022-12-08

**Authors:** Funmilola Olanike Wuraola, Olalekan Olasehinde, Matteo Di Bernardo, Akinbolaji A Akinkuolie, Adewale O Adisa, Adewale A Aderounmu, Tajudeen O Mohammed, Oluwatosin Z Omoyiola, Thomas P Kingham, Olusegun I Alatise

**Affiliations:** 1 Department of Surgery, Obafemi Awolowo University, Ile-Ife, PMB 13, Nigeria; 2 Department of Surgery, Obafemi Awolowo University Teaching Hospitals Complex, Ile-Ife, Nigeria; 3 African Research Group for Oncology, Ile-Ife, Nigeria; 4 Department of Morbid Anatomy and Forensic Medicine, Obafemi Awolowo University Teaching Hospitals Complex, Ile-Ife, Nigeria; 5 Memorial Sloan Kettering Cancer Center, NY, USA

**Keywords:** breast cancer, elderly, low middle income country

## Abstract

**Background:**

Breast cancer in the elderly population has not been evaluated in the Nigerian context. With the rising incidence of breast cancer and the changing demographics, it is likely that an increasing number of elderly patients will be managed in the coming years in Nigeria. This review describes the clinicopathological profile of elderly patients with breast cancer in a Nigerian database.

**Method:**

From a prospective institutional database, elderly patients (65 years and above) managed for breast cancer over a 9-year period were reviewed. Details of their socio-demographic characteristics, patterns of presentation, pathology, treatment and outcome were obtained and analysed.

**Results:**

Of the 607 patients managed during the study period, there were 87 older patients accounting for 14.3% of the total. There was a progressive rise in the number of patients with breast cancer towards the latter part of the study. Expectedly, they were all post-menopausal, with their ages ranging from 65 to 92 years, with a mean of 71 ± 6.58 years. Systemic hypertension was the commonest co-morbidity (29.8%). The mean tumour size at presentation was 10 cm, with the majority presenting with stage 3 disease. Invasive ductal carcinoma was the predominant histological type 83 (95.4%); 44.4% of those who had immunohistochemistry were oestrogen receptor-positive. Approximately half underwent mastectomy (52.8%), 63 (72.4%) had chemotherapy, 8 (44.4%) had hormonal therapy and only 6 (6.9%) had combined multimodal therapy in addition to surgery. Overall 5-year survival was 42.1%.

**Conclusion:**

The pattern of presentation and outcomes of care in this elderly cohort is similar to the general population. Early presentation and use of multimodal treatment is still the mainstay of survival.

## Introduction

Breast cancer is the most common female malignancy and one of the leading causes of cancer deaths globally [1–3]. Low and middle-income countries have an unfair share of the burden of breast cancer with a higher mortality incidence ratio compared to high-income countries [4, 5]. Over the coming years, breast cancer incidence has been projected to increase with a significant proportion of cases coming from low middle income countries (LMICs). More concerted efforts are therefore required to change the poor breast cancer narrative in LMICs.

Much of the data available on breast cancer in many LMICs, particularly in sub-Saharan Africa (SSA) largely depicts events around young women or those in their middle age who constitute the vast majority. The majority of breast cancer patients in many parts of SSA are in their fourth or fifth decades of life [5, 6]. They also tend to exhibit a more aggressive biological behaviour with a high prevalence of triple-negative cancers compared to Caucasian cohorts [7]. Some authors report that the relatively younger age pattern observed in these settings may be an epidemiological issue indicative of the shorter life expectancy in many African countries [8]. Despite younger patients being in the majority, a proportion of patients are seen in the elderly age bracket.

Very little is however known about breast cancer in the elderly in SSA as a distinct group in terms of the pattern of presentation, challenges in management and treatment outcomes. The peculiarities of patients in this age group in terms of comorbidities and other age-related changes might affect the pattern of presentation and treatment in this category of patients. As population dynamics change over time, it will be important to have data capturing this population of patients as a distinct entity.

This study profiles the pattern of presentation and treatment outcomes of breast cancer in elderly patients and also compares with the younger age group from a Nigerian institutional breast cancer database.

## Methods

This was a retrospective review of a prospectively developed electronic breast cancer database at a Nigerian tertiary institution. The data period covers 2010 and 2018. Elderly patients were defined as those 65 years and older at the time of presentation. Ethical clearance was obtained from Obafemi Awolowo University Teaching Hospitals’ ethical committee.

### Sociodemographic data and risk factors

All the participants had their socio-demographic data such as age, gender, educational status and socioeconomic status documented.

Risk factors for breast cancer such as family history, hormonal profile and exposure were documented.

### Clinicopathological details

Clinical, radiological and histopathological details were obtained. This includes the duration of symptoms, tumour size, physical examination findings, imaging, histopathology, immunohistochemistry (IHC) and tumour grade and staging.

### Treatment and follow-up

The treatment details captured include neo-adjuvant and adjuvant chemotherapy, surgery, endocrine therapy and targeted therapy. The following patients or their next of kin on this database were called by a dedicated research assistant every 3 months to determine their status. Patient and/or next of kin receives calls as long as they are alive. Follow-up status was designated as alive and well, alive with disease, dead or unknown. Patients with unknown status were either lost to follow-up or defaulted. Patients were considered lost follow-up if they completed their treatment but were no longer reachable with two missed follow-up contacts. Defaulters are defined as those who did not complete their treatment and were unreachable.

### Data analysis

Details of their socio-demographic characteristics, the pattern of presentation, treatment and outcome were obtained, analysed and presented as descriptive statistics in form of frequency tables and charts comparing both age groups.

A detailed descriptive analysis of patients 65 years and above was done, and the data were compared to the patients below 65 years (18–64 years).

The time to mortality was analysed using the Kaplan–Meier curve. Statistical analyses were done using the software Statistical Package for Social Science (SPSS) version 24 (IBM SPSS Inc.).

## Results

### Clinical characteristics

Within the period under review, a total of 607 patients were managed with breast cancer. Eight-seven (14.3%) of them are aged (65 years and above) and 520 (85.7%) were below 65 years. The study population was predominantly females 81 (93.1%). In the patient group under 65 years, 515 (99%) were females. The age range was from 65 to 92 years with a mean age of 71 ± 6.58 years. While the mean age for <65 years is 46.6 ± 9.26. All the females above 65 were post-menopausal, with average parity of six children. Only one patient had a family history of breast cancer in a first-degree relation, four (4.5%) drank alcohol and there was no history of cigarette smoking recorded in any of the patients. Hypertension was the commonest co-morbidity and was seen in 26 (29.9%) of patients, followed by diabetes mellitus (5.7%). The median duration of symptoms was 6 months with a range of 1–84 months. All patients presented with self-detected breast mass, with clinical measurements ranging from 2 to 26 cm in the widest diameter – Four (4.6%) patients presented with bilateral breast cancer. Nearly half of the patients, 43 (49.4%) presented with Stage III disease with only 2 (2.3%) patients presenting with stage 1 disease. A total of 60 (68.9%) had stages III and IV disease.

### Histopathological characteristics

Invasive ductal carcinoma was the commonest histological type in 83 (95.4%), followed by mucinous carcinoma in 4 (3.6%). The Nottingham grading was grade 1 in 51 (58. 6%) while grades 2 and 3 accounted for 18 (41.1%) patients in each group.

IHC was done in 18 (20.7%) patients with 8 (44.4%) of them being oestrogen, progesterone positive and 6 (33.3%) Her-2neu positive and 6 (33.3%) triple negative.

### Treatment

Chemotherapy (neo-adjuvant (65.5%) or adjuvant (32.2%)) was administered to 63 (72.4%), the majority had anthracycline-based combinations and surgery was performed on 47 (54%). Modified radical mastectomy was performed in 42 (48.3%), while 5 (5.7%) had a simple mastectomy. Six (6.9%) patients had radiotherapy to the chest wall. Forty-five (51.7%) had both chemotherapy and surgery done. Hormonal therapy was administered to 8 (9.2%) patients. Overall, only 6 (6.9%) had surgery, radiotherapy and chemotherapy.

### Outcomes

Follow-up data showed that 47 (54.0%) patients were alive, 30 (34.0%) were dead, 5 (5.7%) defaulted and 5 (5.7%) had an unknown status.

Of the 46 patients that had a mastectomy, 12 (26%) had locoregional recurrence, and 50% recurred within the first year of surgery. The anterior chest wall was the commonest site of recurrence seen in 10 (76.9%) of patients.

**Figure 1.**
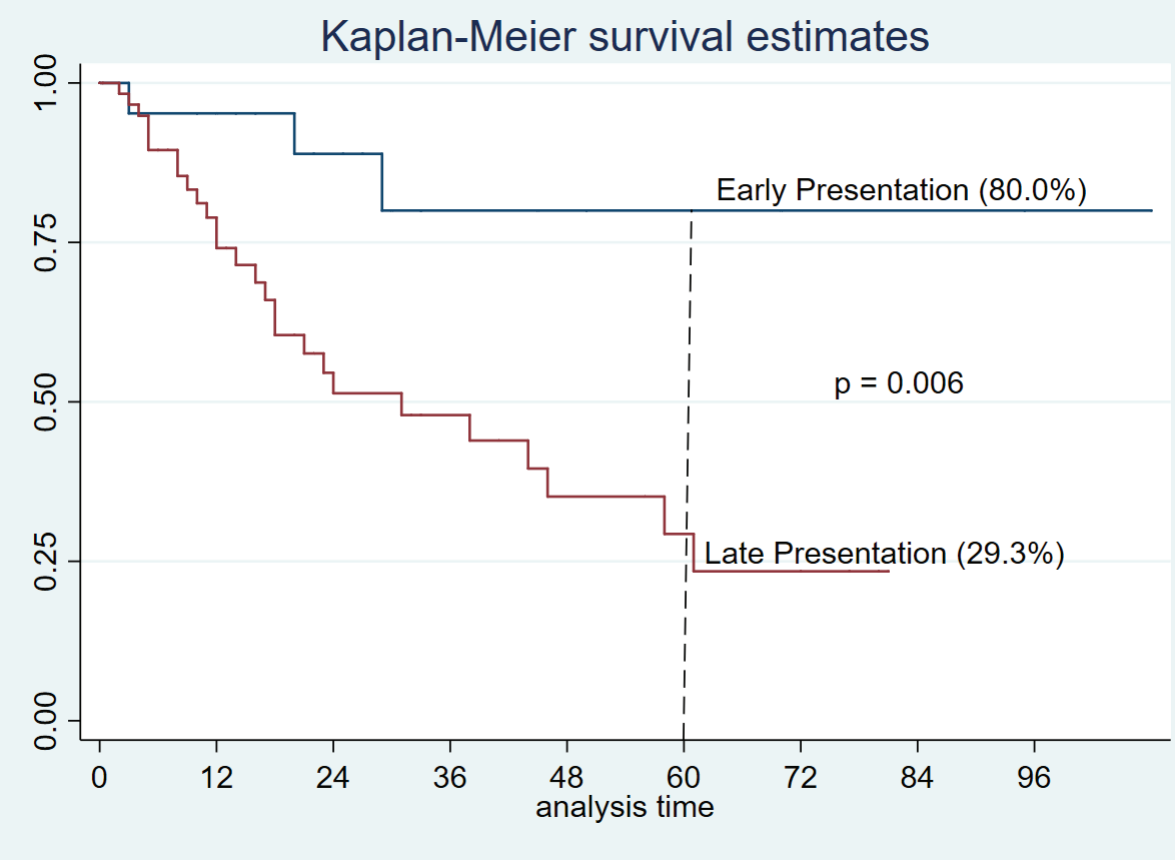
Survival curve of time to death from presentation showing 5-year survival in older patients.

The mortality rate was 34.4% (30 patients), 47 (54.0%) patients were alive and 10 (11.5%) lost to follow-up. Survival was based on 77 patients with completed data, the cumulative 5-year survival was 42.1% and stage at presentation was significantly associated with survival (*p* = 0.001, Figure 1). (Early presentation is stage I and II while late presentation is stage III and IV.)

### Comparison of elderly with younger patients

The pattern of presentation, pathological characteristics and treatment administered were not significantly different between the two age categories. The risk factors such as parity between the two groups were significant with a *p*-value of 0.001, while the use of hormonal contraceptives *p*-value of 0.274, family history of breast cancer *p*-value of 0.144 and alcohol consumption was 0.343 all are not statistically significant. The IHC status is not statistically significant (See Tables 1–3). The 5-year survival for the elderly category was 42.1% while for those <65 years was 49.2%. This difference was however not statistically significant.

## Discussion

The older population constitutes a distinct group with peculiar physiological and disease-related characteristics which may modify the approach to their care. Their needs as well as expectations following breast cancer treatment may also vary slightly among younger patients. Generally, there is some data bias towards younger patients regarding breast cancer management [9]. Older patients are often excluded from clinical trials which form the basis for designing practice guidelines. Although in the minority, the current demographic change in many LMICs suggests that more older patients will require breast cancer management in the near future [10]. This study which focused on older women managed in a Nigerian tertiary hospital is novel and serves as a reference on which further studies on older patients with breast cancer can be based.

**Figure 2.**
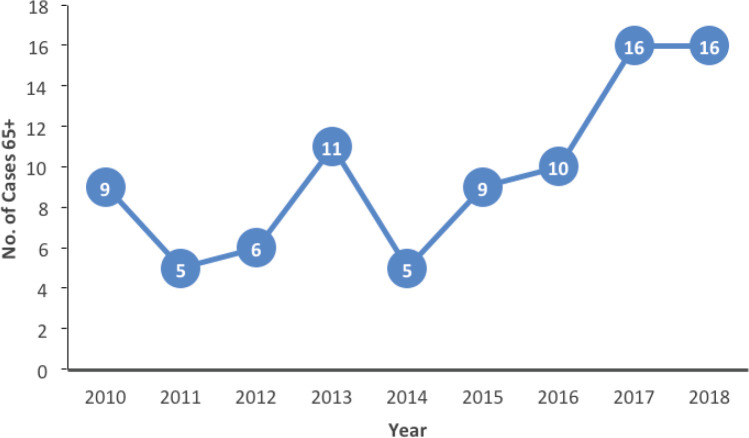
Number of patients per year of presentation.

Older patients are sub-classified into categories (youngest old, middle old and oldest old) [11]. The majority of patients in this series belonged to the youngest old category. Higher-income countries report a greater number of patients in the older categories compared to our findings. This difference in the epidemiological pattern may be a reflection of the disparity in life expectancy between high and low-middle-income countries. In this series, older patients represented only 14% of the entire number of breast cancer patients managed in the study period. This is much lower than reports from high-income countries where older patients (70 years and older) constitute about 35% of the total number of breast cancer patients in some series [12, 13]. This difference might not be unconnected to the poor access to screening and diagnostic services typical of many LMICs like Nigeria [14, 15]. Although the overall proportion of older patients was low, we observed a rising trend in the number of older patients in the latter period of the study period (Figure 2). This may be related to increased awareness as well as improvements in diagnostic services. If this trend continues, a more sizeable number of older patients may require breast cancer care in the coming years.

The peculiarities of breast cancer management in the older age groups are both related to tumour biology and to age-related comorbidities typically found in this subset of patients. Co-morbidities can affect the decision to offer appropriate treatment such as surgery, chemotherapy or targeted therapy. Surgery might be avoided in some patients with severe comorbidities due to the stress on the heart. Some chemotherapeutic agents also need to be avoided or reduced due to their effects on cardiac function, all these might lead to the undertreatment of elderly patients with breast cancer with potential effects on the outcome. Besides systemic hypertension, very few patients had severe comorbidities in this series [16–18]. There is the possibility of a selection bias given the relatively poor health care system which might have limited patients with more severe comorbidities from accessing care. The relatively younger age of patients in this cohort might also explain the lower prevalence of severe comorbidities [19].

It is known that older patients tend to have low-grade diseases and higher rates of oestrogen receptor positivity. Relative to younger patients, this review did not show a difference in the grade of disease and oestrogen receptor status in the elderly cohort. This finding should however be further evaluated given that IHC was done in only a few patients. The limited number of IHCs done is a reflection of the disparity in cancer management in LMIC, where access is still a major issue.

This study reports a 5-year survival of 42% overall. When analysed in layers, however, patients who presented with the early disease had almost double this rate (Figure 1). This finding supports the need to extend screening and diagnostic services to elderly patients who are generally known to be underserved. The fewer number of patients who received care (surgery, chemotherapy and endocrine therapy) relative to the number of potentially treatable patients is concerning. It is generally known that older patients tend to be undertreated largely due to frailty and co-morbidities [9, 13, 20, 21]. However, in this cohort, co-morbidity is not the reason for undertreatment, because very few elderly patients have co-morbidities, it’s rather due to other reasons such as lack of financial access to care, sociocultural factors and belief in alternative remedies are some of the factors that have been previously identified as barriers to complete breast cancer care in this setting.

From the foregoing, this study highlights the possibility of a rising number of older patients requiring breast cancer care in the coming years given the data seen at this centre in Nigeria. The favourable biological pattern observed in this cohort can only be maximised provided there is access to prompt diagnostic services and treatment. This study provides background data on this subject on which further studies can be built.

We recommend that to improve breast cancer care in this age group, a holistic approach is important to foster early detection, prompt diagnosis and adequate treatment. This will involve repeated advocacy and improved health policies.

### Limitations

The number of elderly patients in this cohort is few; however, this shows the difference in breast cancer incidence by age in Nigeria as compared to other parts of the world. Also, the number of patients that received complete treatment is small and this might also affect the outcome.

## Conclusion

Breast cancer in the elderly in Nigeria is not different in presentation to younger people and its management is also facing similar challenges.

## Conflicts of interest

The authors declare no conflicts of interest.

## Funding

No funding was given for this study.

## Figures and Tables

**Table 1.** Clinicopathology.

Variable	<65 *N* (%)	65+ *N* (%)	*p*
Mean age (M ± SD)	46.6 ± 9.26	71.0 ± 6.58	0.001
Mean tumour size (M ± SD) Mean duration of symptoms	10.5 ± 6.64 10.02 ± 13.07	9.1 ± 5.40 10.2 ± 14.4	0.097
Stage of disease			
Stage 1	6 (1.2%)	1 (1.1%)	0.14
Stage 2	72 (13.8%)	20 (22.9%)	
Stage 3	324 (62.3%)	45 (51.8%)	
Stage 4	106 (20.4%)	17 (19.6%)	
Unknown	12 (2.3%)	4 (4.6%)	
Histopathological type			

**Table 2.** Compare the age group by ER status, PR status and HER2 status.

		Age				
		<65		65+		Statistics
		*N*	%	*N*	%	Chi-square (*p*-value)
ER status	Negative	68	60.2	10	55.6	0.138 (0.711)
	Positive	45	39.8	8	44.4	
PR status	Negative	79	69.9	10	55.6	1.469 (0.225)
	Positive	34	30.1	8	44.4	
HER status	Equivocal	5	4.4	2	11.1	
	Negative	71	62.8	10	55.6	1.180 (0.554)
	Positive	37	32.7	6	33.3	

**Table 3.** Treatment.

Variable	<65 *N* (%)	65+ *N* (%)	*p*-value
Surgery Yes No	257 (49.4%) 263 (50.6%)	47 (54%) 40 (46%)	0.43
Radiotherapy Yes No Unknown	41 (7.9%) 469 (90.2%) 10 (1.9%)	6 (6.9%) 81 (93.1%) 0 (0%)	0.20
Chemotherapy Yes No	374 (71.9%) 146 (28.1%)	64 (73.6%) 23 (26.4%)	0.75
